# Vestibular effects on cerebral blood flow

**DOI:** 10.1186/1471-2202-10-119

**Published:** 2009-09-23

**Authors:** Jorge M Serrador, Todd T Schlegel, F Owen Black, Scott J Wood

**Affiliations:** 1 Harvard Medical School, Beth Israel Deaconess Medical Center, Boston, MA, USA; 2 NASA Johnson Space Center, Houston, TX, USA; 3 Neurotology Research, Legacy Health System, Portland, OR, USA; 4 Universities Space Research Association, Houston, TX, USA; 5 National University of Ireland Galway, Galway, Ireland

## Abstract

**Background:**

Humans demonstrate a number of unique adaptations that allow for the maintenance of blood pressure and brain blood flow when upright. While several physiological systems, including cerebral autoregulation, are involved in this adaptation the unique role the vestibular system plays in helping to maintain brain blood flow is just beginning to be elucidated. In this study, we tested the hypothesis that stimulation of the vestibular system, specifically the otoliths organs, would result in changes in cerebral blood flow.

**Results:**

To test our hypothesis, we stimulated the vestibular organs of 25 healthy subjects by pitch tilt (stimulates both canals and otoliths) and by translation on a centrifuge (stimulates otoliths and not the canals) at five frequencies: 0.5, 0.25, 0.125 and 0.0625 Hz for 80 sec and 0.03125 Hz for 160 sec. Changes in cerebral flow velocity (by transcranial Doppler) and blood pressure (by Finapres) were similar during both stimuli and dependent on frequency of stimulation (P < 0.01). However, changes in cerebral blood flow were in opposition to changes in blood pressure and not fully dependent on changes in end tidal CO_2_.

**Conclusion:**

The experimental results support our hypothesis and provide evidence that activation of the vestibular apparatus, specifically the otolith organs, directly affects cerebral blood flow regulation, independent of blood pressure and end tidal CO_2 _changes.

## Background

To maintain arterial pressure when upright, humans must respond to a translocation of blood from the upper into the lower body. Responses to this translocation include baroreflex-mediated increases in heart rate and peripheral vasoconstriction, thus compensating for reduced venous return and minimizing pooling of blood in the lower body. Previous work in animals has demonstrated that sectioning the vestibular nerve to remove information on position relative to gravity results in a dramatic increase in postural hypotension [1].

**Figure 1:**
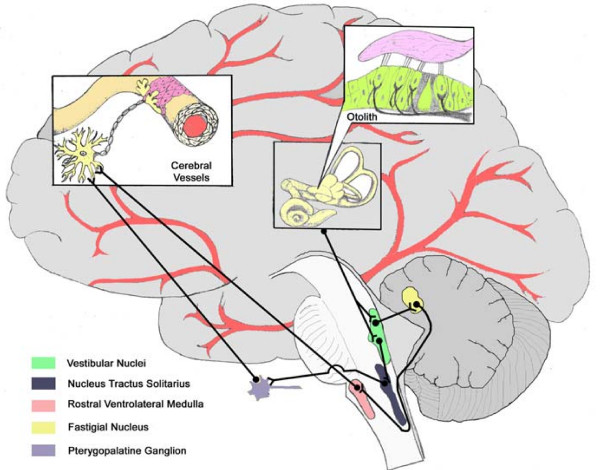
**Vestibular Cerebrovascular Connections**. Anatomical connections demonstrating possible pathways connecting vestibular organs and the cerebral vessels.

While a role for the vestibular system in the autonomic response to the upright position has been documented, there have been no studies demonstrating a direct effect of otolith stimulation on cerebral blood flow. Anatomical evidence in animals demonstrates that neural connections are present between the vestibular nuclei and cerebral vessels through two possible pathways (Figure 1). Connections have been found between the Vestibular Nuclei and the Fastigial Nucleus [2], then to the Rostral Ventrolateral Medulla [3], followed by vasodilatory connections to the cerebral vessels [4]. Similarly, neurons travel from the Vestibular Nuclei to the Nucleus Tractus Solitarius [5] and then to the Pterygopalatine Ganglion [6], resulting in cerebral vasodilation [7-9]. However, the role these connections play in human postural adjustments remains to be determined.

Caloric vestibular stimulation in humans that activates the semicircular canals, involved with detection of movement (i.e. angular acceleration), has been found to increase blood flow in the basilar [10] and middle cerebral arteries [11] as well as the parietal lobe [12] while decreasing flow in the posterior cerebral artery [11]. However, it remains unclear whether these changes were due to functional activation of vestibular and other centers, rather than general vascular changes. We propose that otolith afferents that signal both magnitude and change in orientation relative to gravity will be more critical than canals for vestibulo-cerebrovascular interactions.

Our group has previously found that subjects exposed to 30 minutes of hypergravity demonstrate impaired cerebral blood flow regulation that returned to normal upon assumption of the upright posture [13]. Furthermore, this impairment was found to correlate with non-invasive measures of otolith sensitivity, providing indirect evidence of a role for otolith activation. Similarly, using head down neck flexion, we found a modulation of cerebrovascular resistance (CVR) [14] that may have been due to otolith activation. Finally, in subjects that developed nausea during centrifugation, cerebral blood flow was reduced almost two minutes prior to actual nausea [15]. Since centrifugation was performed in the dark with no visual cues, these data suggest a role for vestibular inputs in affecting the cerebrovasculature.

In the present study, we examined the affects of vestibular stimuli on cerebral blood flow by utilizing: 1) prolonged variable-radius centrifugation (to elicit otolith activation without stimulating the semicircular canals); and 2) a pitch tilt stimulus about an Earth-horizontal axis (to elicit cues from both the otolith organs and the semicircular canals). This same paradigm has been useful in previous studies to elucidate the relative roles of otoliths and canals on ocular responses and motion perception across broad range of stimulus frequencies [16]. Since otolith cues are similar in both variable radius centrifugation and tilt paradigms while canals cues are not, similar frequency dependent changes in cerebral blood flow would be predicted for both paradigms if the effect was primarily due to otolith rather than canal activation.

Canal afferent sensitivity between 0.1 and 1 Hz is fairly constant with little phase error, but begins to drop off at lower frequencies [17]. Regular otolith afferents, on the other hand, are sensitive to even constant tilts, while irregular afferents exhibit an increase sensitivity and less phase error with increasing frequency; i.e., responding to rate of change in linear acceleration [18]. Therefore, frequency dependent effects are also helpful to elucidate the role of tilt-mediated (static) versus translation-mediated (dynamic) otolith afferents [19].

## Methods

### Subjects

Twenty four healthy, non-smoking subjects (29 ± 9 years, 72 ± 12 kg, 173 ± 9 cm, 11 females) were recruited. The study protocol was approved by the Legacy Health Systems Institutional Review Board and all subjects provided informed written consent.

### Experimental Protocol

#### Instrumentation

Beat-by-beat blood pressure was measured by photoplethysmographic cuff on a finger (Finapres, Ohmeda, CO, USA). End-tidal CO_2 _was sampled from expired air via a nasal catheter (Puritan-Bennett, Wilmington, MA, USA). Cerebral flow velocity (CFV) in the middle cerebral artery was obtained by transcranial Doppler (MultiDop T, DWL, Germany) as previously described [20]. All physiologic signals were digitized at 500 Hz (Windaq, Dataq Instruments, OH, USA). Cerebrovascular reactivity was assessed during 3 min of resting ventilation, inspiration of 8% CO_2_, 21% O_2_, balance nitrogen for 2 min and mild hyperventilation for 2 min.

#### Tilt Protocol (Figure 2A)

**Figure 2:**
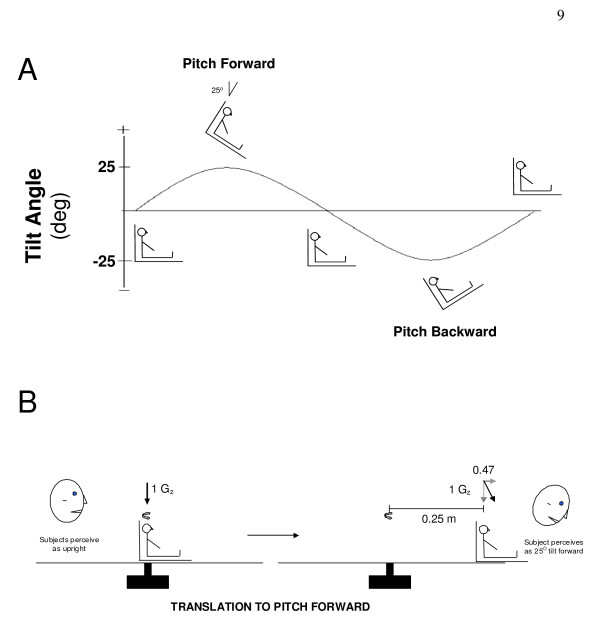
**Protocol Overview**. A) Diagrammatic representation of the relative chair position over the course of each cycle. B) Representation of movement into the pitch-forward position during centrifugation. Subjects were oscillated back at forth and 5 frequencies to produce sufficient centripetal acceleration to create corresponding gravitoinertial acceleration (GIA) that produced similar perceptions of tilt to the pitch-tilt chair protocol.

Subjects were seated upright in a high-torque hydraulic-powered tilt chair capable of delivering controlled angular accelerations of > 1,000°/s^2^. Subject position was adjusted to align the interaural axis near the rotation axis and Reid's plane aligned with Earth horizontal. The vestibular stimulation consisted of rotating the chair in the pitch plane +/- 25° with a sinusoidal motion profile. Thus subjects went from sitting upright to pitch forward at a 25° angle followed by being pitched backwards at a 25° angle and then returned to the upright position. This motion profile was done at five frequencies: 0.5 Hz (40 cycles); 0.25 Hz (20 cycles); 0.125 Hz (10 cycles); 0.0625 Hz (5 cycles) and 0.03125 Hz (5 cycles) with 80 s rest in-between stimulation frequencies. Holding the tilt displacement across frequencies meant the velocity ranged between ± 5 and ± 79 deg/s, while the equivalent linear acceleration due to tilt remained ± 4.1 m/s^2 ^across all frequencies.

#### Centrifugation Protocol (Figure 2B)

The rationale for this protocol is that once subjects are rotating at a constant rotational velocity, they no longer experience angular acceleration. We can then create a linear outward acceleration by moving subjects off of the centre. Subjects were seated upright over the centre axis of rotation on a short-arm centrifuge device driven by direct-drive motor (80 ft lb). Subjects were restrained so as to minimize motion of their torso, legs and head during centrifugation and all centrifugation was performed in the dark to eliminate visual cues of orientation relative to gravity. Subjects were rotated about an earth-vertical axis for at least 5 minutes, allowing the horizontal canal cue to decay, before proceeding with sinusoidal translations in the anterior-posterior (AP) direction. The resultant acceleration in the saggital plane is a sum of the radial acceleration due to chair translation and the centripetal acceleration due to off-center rotation. Both radial and centripetal acceleration are in phase with chair displacement peaking at maximal chair displacement. The chair displacement at each frequency was adjusted so that the peak resultant linear acceleration was 25 deg off-vertical to match the tilt stimuli described above. Although there was also a Coriolis acceleration in the orthogonal (lateral) direction, these effects appeared to be negligible on otolith-mediated eye movements in a previous study [16].

The protocol consisted of:

• 3 min baseline

• Acceleration at 25°/s^2 ^to constant rotational velocity of 250°/sec over center of rotation for 5 min

• Translation of the chair along a track that is spinning at constant rotational velocity. The chair was translated back and forth in the pitch plane at 5 frequencies: 0.5 Hz (+/- 6.2 cm); 0.25 Hz (+/- 8.4 cm); 0.125 Hz (+/- 9.2 cm); 0.0625 Hz and 0.03125 Hz (+/- 9.4 cm) using the same cycles and rest as tilt. The frequency order was randomized across subjects and identical to the tilt protocol.

• Deceleration to complete stop at 25°/s^2^

Six subjects that became nauseated were not included in the analysis for either the centrifugation or tilt protocols

### Data Processing and Analysis

Post-processing was done using custom-written MATLAB scripts (The Mathworks, Natick, MA). CVR was calculated from brain level blood pressure (derived using hydrostatic correction) and CFV. Cerebrovascular reactivity was determined by a linear fit of beat-by-beat cerebral flow velocity with associated end-tidal CO_2 _values after incorporating the known 6 s time delay between end-tidal CO2 changes and associated CFV response[21] The effects of stimulus (Tilt vs. Centrifugation) and Frequency on CFV, arterial pressure, end tidal CO_2_, and CVR were assessed using a repeated-measures General Linear Model (SPSS, Chicago, IL, USA). To compare frequency of stimulus, data were collapsed so the same number of data points were available for each cycle (i.e. for 0.5 Hz, 8 data points were derived from the average of each 0.25 sec period; for 0.03215 Hz, 8 data points were derived from each 4 sec period). To examine the role of position vs. the rate of change in position (velocity) on the CFV response, these variables were correlated to CFV at each frequency. Data are presented as mean ± **SD**.

## Results

**Figure 3:**
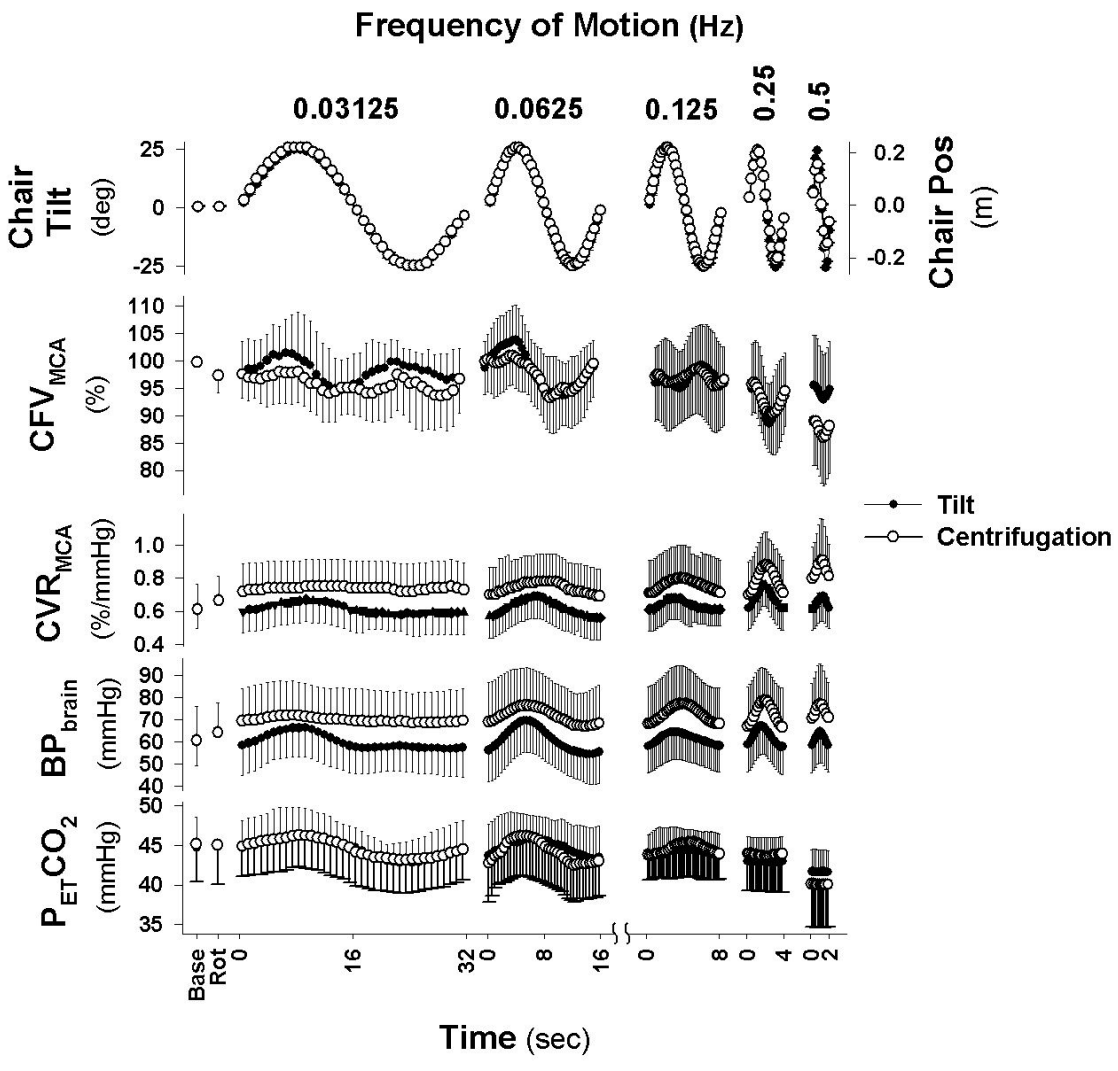
**Cerebral Blood Flow Response to Vestibular Stimulation**. Response of subjects to five frequencies of stimulation averaged over 40 cycles for 0.5 Hz, 20 cycles for 0.25 Hz, 10 cycles of 0.125 Hz, and 5 cycles of 0.0625 Hz and 0.03125 Hz. Cerebral flow velocity (CFV) in the middle cerebral artery was affected at all frequencies during sinusoidal translation in the pitch plane (providing a pitch tilt stimulus) while being rotated at 250 deg/sec (CEN - Centrifugation) as well as sinusoidal ± 25 degree pitch tilt (TILT). Base represents mean of 3 min baseline while sitting quietly in both conditions and Rot represents three minute average while rotating on center during centrifugation.

Table 1 demonstrates that the baseline values for each dependent measure were not significantly different between the tilt and centrifuge test sessions. Both centrifugation and tilt resulted in a modulation of CFV as measured by transcranial Doppler that were linked to stimulation frequency (Figure 3). CFV responses were dependent on frequency of stimulation (P < 0.01) and demonstrated significant changes within each frequency cycle (P < 0.01) that differed by frequency (Frequency × Cycle interaction, P < 0.01). While CFV during centrifugation at 0.5 Hz tended to be lower than during tilt (P = 0.07), the change within the cycle was very similar. The CFV responses shown in Figure 3 may have been due to factors unrelated to the otoliths, such as driving pressure or changes in arterial CO_2 _levels. For example, there was a significant effect on blood pressure at brain level of both frequency of stimulation (P < 0.01) and position within cycle (P < 0.01) that differed by frequency (Frequency × Cycle interaction, P < 0.01). In addition, blood pressure was significantly higher during centrifugation than tilt at all frequencies (P < 0.01). However, the patterns of the blood pressure and CFV changes were different. For example, as shown in the left panel (0.03125 Hz) of Figure 3, CFV increased during both the +25° and -25° tilt positions whereas blood pressure increased only during the +25° tilt position. Similarly at 0.25 and 0.5 Hz, blood pressure increased during the first half of the cycle (i.e. moving from upright to pitch forward and back to upright) at the same time CFV decreased. Thus CFV was decreasing even though driving pressure was increasing. Thus, while blood pressure consistently increased somewhere between a quarter (slowest frequency) and half way into the cycle (fastest frequency), CFV demonstrated bimodal peaks at the slowest frequency and decreases at the fastest frequency (Figure 3). This would again suggest a disparity in the response of the two variables. These data therefore suggest that otolith activation at various frequencies likely directly affects cerebral blood flow.

**Table 1:** 

	**Tilt**	**Centrifugation**	
	**Upright** **Seated** **Baseline**	**Upright** **Seated** **Baseline**	**Rotation** **On Centre**
Cerebral Flow Velocity (cm/s)	66.2 ± 10.3	67.9 ± 13.3	66.1 ± 12.9
Mean Arterial Pressure (mmHg)	80.2 ± 11.6	81.0 ± 14.6	84.7 ± 12.6
Heart Rate (bpm)	67.0 ± 14.9	66.0 ± 13.9	68.0 ± 14.9
End tidal CO_2 _(mmHg)	44.7 ± 3.6	45.1 ± 4.6	45.0 ± 5.0

Changes in end tidal CO_2_, an indicator of arterial CO_2_, were similarly disparate from those in CFV. First, end tidal CO_2 _was also affected by both frequency of stimulation (P < 0.01) and position within cycle (P < 0.01) that differed by frequency (Frequency × Cycle interaction, P < 0.01). In addition end tidal CO_2 _was significantly lower during centrifugation with translation at 0.5 Hz. However, examination of individual frequencies showed differing patterns. For example, at 0.03125 Hz, end tidal CO_2 _increased at +25° and decreased at -25°, while CFV increased at both +25° and -25°. Similarly at 0.25 and 0.5 Hz, end tidal CO_2 _did not change within the cycle, while CFV decreased significantly. These data demonstrate that changes in end tidal CO_2 _cannot completely explain position-related changes in CFV.

While there was no significant difference in responses to either sinusoidal tilt or translation during centrifugation at the four slowest frequencies, CFV was 6.8 ± 0.3% lower during centrifugation. Since end tidal CO_2 _was also 1.6 ± 0.3 mmHg lower, based on the cerebrovascular reactivity of 2.6%/mmHg, approximately two thirds of the 6.8% decrease could be explained by the centrifugation-related hypocapnia.

Changes in CFV are likely mediated through changes in CVR. Since CFV is normally regulated to maintain flow relatively constant in the face of changing perfusion pressure--a phenomenon known as cerebral autoregulation [22] -- changes in CVR could result from changes in blood pressure (autoregulation) or changes in otolith afferent activity (vestibular). Figure 3 demonstrates that changes in CVR within the motion cycle were similar to the changes in blood pressure, suggesting an autoregulatory response. However, if CVR changes were solely autoregulatory in nature, CFV would have remained constant throughout motion. The fact that CFV was changing throughout the cycles indicates CVR changes were not sufficient to maintain flow indicating that a non-autoregulatory component was influencing CVR and causing changes in flow.

**Figure 4:**
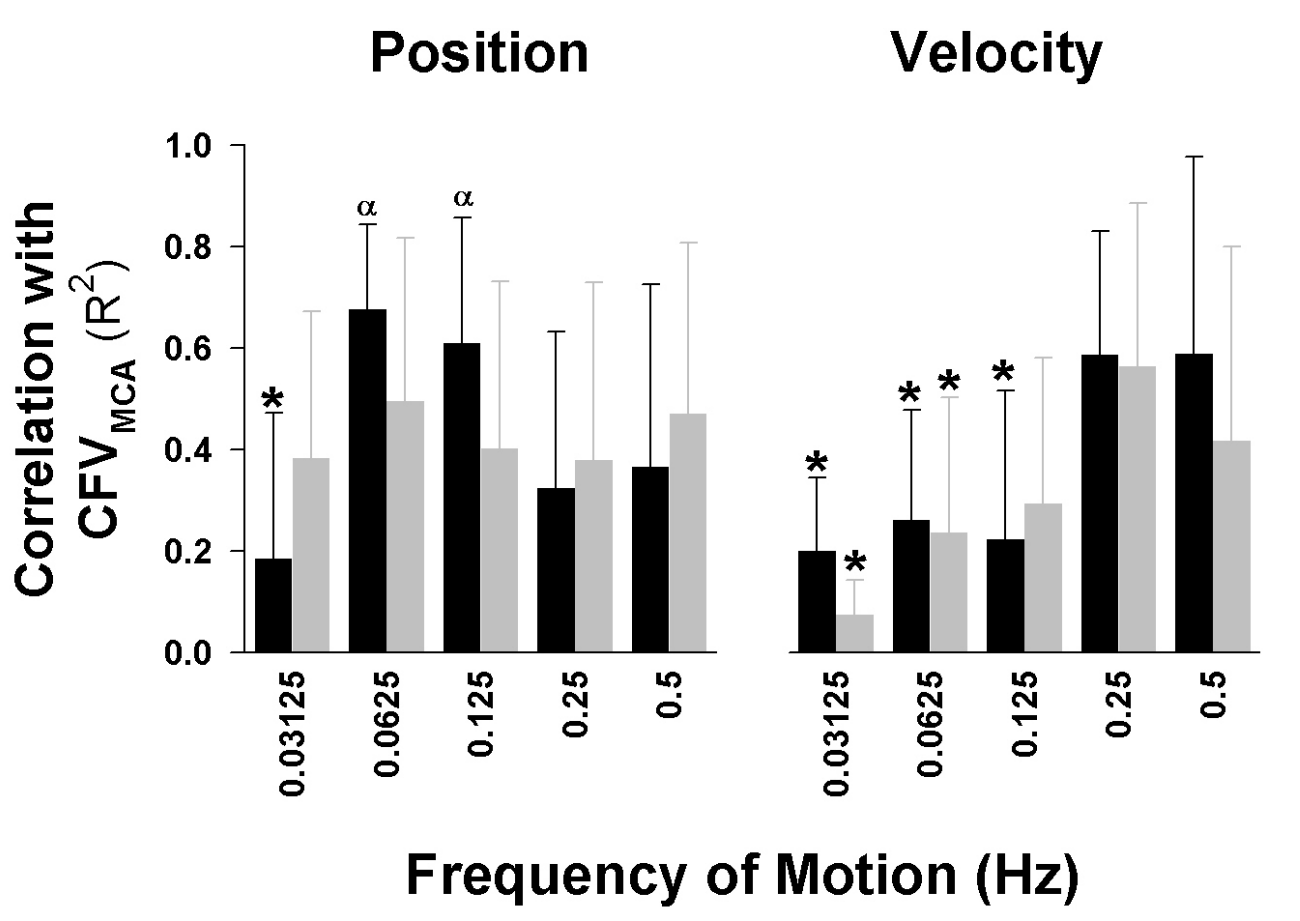
**Relationship between Vestibular Inputs and Cerebral Blood Flow Response**. Correlation between position (left panel) or velocity (right panel) and cerebral flow velocity (CFV) at the various frequencies. Changes in CFV were correlated to velocity only at the highest two frequencies for both tilt (TILT) and centrifugation (CEN). In contrast, changes in CFV were correlated to position at all frequencies for centrifugation. During tilt, changes were strongly correlated at 0.0625 & 0.125 Hz, with lower values at the higher and lowest frequencies. *, indicates significant difference from 0.5 Hz (P < 0.05); α, indicates significant difference from velocity correlation at that frequency (P < 0.05).

To further explore the role of frequency in the response of CFV to vestibular activation, we examined the correlation between CFV and either chair position or velocity of motion throughout the cycle (Figure 4). As can be seen in Figure 4, during tilt, changes in CFV in the low frequency range (0.0625-0.125 Hz) were especially correlated to position (left panel), whereas those in the high frequency range (0.25-0.5 Hz) were especially correlated to the velocity of motion (right panel). Correlations between CFV and the velocity of motion were significantly lower in the 0.03125-0.125 Hz ranges compared to the 0.25-0.5 Hz ranges for both tilt and centrifugation. A generally opposite pattern was demonstrated for position (left panel), where correlations between CFV and position were ~0.6 for tilt at 0.0625 & 0.125 Hz, decreasing to ~0.4 at the higher frequencies. Interestingly, at the lowest frequency for position, the correlation was only 0.2.

## Discussion

Our overall results are consistent with vestibular activation at different frequencies of motion causing changes in cerebral blood flow that are in part independent of changes in both mean arterial pressure and end tidal CO_2_. We believe that these data are the first to show that responses in CFV during centrifugation and tilt stimuli cannot be fully accounted for by change in either mean arterial blood pressure and/or end tidal CO_2_. Since similar findings were obtained during both centrifugation involving otolith cues and tilt stimuli involving otolith and canal cues, the changes in CFV are therefore likely dependent on otolith stimulation in particular.

Interestingly, sinusoidal pitch tilt and centrifuge stimuli had two main effects. First, changes in CFV in the low frequency range were generally significantly correlated to position. Regular otolith afferents across this frequency range are known to have constant sensitivity and small phase errors [18]. This would suggest that otolith inputs of tilt angle were the predominant underlying factor in CFV changes at these frequencies. However, it remains unclear why the slowest frequency of motion during tilt (0.03125 Hz) produced the lowest correlations between position and CFV. One possible explanation is that at the lowest frequency the response depended on absolute tilt angle (i.e. irrespective of direction) rather than relative angle (i.e. forward tilt vs. backward tilt). However, correlating CFV to absolute tilt at 0.03125 Hz only increased the R^2 ^to 0.25 ± 0.5. The changing correlations during tilt may reflect the influence of vertical canals indirectly through canal-otolith integration at the site of the vestibular nuclei. Another explanation may be that during tilt at very low frequencies (i.e. 0.03125 Hz), other somatosensory cues also influence the CFV response, thus masking vestibular effects. These non-vestibular gravitational cues may have low-pass filtered dynamics [23] that may differ between tilt and centrifugation paradigms. In a similar fashion, cardiovascular mechanisms, such as the baroreflex, may also be dominant at lower stimulus frequencies and sufficient to compensate for orthostatic challenges without feed-forward input from the otoliths.

In contrast, during centrifugation the correlations with position did not change significantly across frequencies. This may have been due to the lack of canal inputs and the fact that the novel experience of centrifugation eliminated normal somatosensory cues which may have masked vestibular inputs.

The second major effect of vestibular stimulation was the direction-dependent effect at frequencies greater than 0.03125 Hz. Movement from pitch forward (i.e. semi-prone) to pitch backward (semi-supine) resulted in increases in CVR and decreases in CFV (Figure 3). It remains unclear why there was a direction-dependent effect of pitch. Cheung et al [24] reported greater postural hypotension during roll vs. pitch tilts, suggesting that cardiovascular responses to tilt may be direction dependent. Similarly we previously found that changes in CFV during head position manipulation differed in the prone vs. supine position [14]. Since the plane of the otolith maculae is tilted back about 20° relative to our upright position (Reid's baseline parallel to Earth-horizontal plane), the pitch forward tilts would bring the otoliths into a position of increased sensitivity whereas the pitch backward would bring them into a position of reduced sensitivity [25]. Thus, the differences in response may be due to differing otolith sensitivity in the two positions.

It is also interesting to note that irregular otolith afferents, which detect change in linear acceleration which is more associated with motion, become increasingly sensitive at higher frequencies [18]. This response is manifested in the oculo-motor correlate that at higher frequencies, linear acceleration produces horizontal eye movements that compensates for retinal slip velocity, whereas at lower frequencies we see ocular counter-roll, associated with tilt response [19]. Another possible explanation for the direction-dependent effect at higher frequencies is that by maintaining the equivalent tilt stimuli constant across frequencies, the velocity amplitude increased as a function of stimulus frequency. Regardless of the exact etiology of these responses, our data are consistent with a vestibular-mediated frequency dependent effect on the cerebral blood flow response to postural position changes relative to gravitoinertial forces.

## Limitations

Previous work has demonstrated that vestibular activation can cause changes in blood pressure [1]. Thus, it might be theorized that the relationship between CFV and chair position was mediated through underlying changes in blood pressure. At the 0.5 Hz frequency, for example, chair oscillations occur every 2 seconds and could cause concomitant changes in blood pressure. When there is an associated change in cerebral perfusion pressure, cerebral autoregulation returns CFV to baseline levels by myogenic dilation or constriction in response to the stretch of the vessel [22]. Since this dilation/constriction takes a few seconds to engage [26], rapid changes in flow could be the result of rapid changes in blood pressure. However, at both the 0.25 and 0.5 Hz frequencies, CFV decreased at the same time blood pressure increased. Since autoregulation serves only to return flow to baseline levels and has not been previously shown to drive diminutions in CFV that oppose the direction of blood pressure, these data seem inconsistent with changes in CFV being driven by those in blood pressure. Similarly, if CFV were just responding to changes in blood pressure (i.e. stretch of vessels), then at slower frequencies, when autoregulation had sufficient time to engage, CFV should have remained relatively constant, but did not. It might also be theorized that a time lag in the autoregulatory response could account for the greater correlation of CFV with velocity at high frequencies and with position at lower frequencies (i.e. at higher frequencies changes in blood pressure were so rapid that autoregulation did not have time to engage and thus changes in CFV were due to changes in pressure). This seems unlikely because CFV was moving in the opposite direction of blood pressure and decreases in CFV were greatest mid cycle at both 0.25 and 0.5 Hz. If this response were due to the time lag in autoregulatory response (~1-3 sec) [26], then the trough should have occurred at the same time in both conditions. Instead the trough occurred at 1 sec in the 0.5 Hz cycle and 2 sec in the 0.25 Hz cycle, in alignment with the motion profile in the respective cycle, indicating again that the change was likely related to the motion profile.

## Conclusion

These findings suggest a role for vestibular inputs in cerebral blood flow regulation. Future work examining this connection in clinical populations might lead to new treatment modalities for cerebral hypoperfusion under a variety of circumstances. For example, with aging there is well documented vestibular loss that might contribute to reduction in global cerebral blood flow. Similarly, patients with orthostatic intolerance could have vestibular impairment that exacerbates cerebral hypoperfusion when upright.

## Authors' contributions

JS and SW conceived the experiment, and together with FOB carried it out; JS designed and carried out the data analysis; JS and SW co-wrote the paper with scientific interpretation input from TS and FOB.
